# A Broadband Phototransistor Based on Three-Dimensional Reduced Graphene Oxide Foam

**DOI:** 10.3390/nano8110913

**Published:** 2018-11-06

**Authors:** Yifan Li, Yating Zhang, Yu Yu, Zhiliang Chen, Lufan Jin, Mingxuan Cao, Haitao Dai, Jianquan Yao

**Affiliations:** 1 Key Laboratory of Opto-Electronics Information Technology (Tianjin University), Ministry of Education, School of Precision Instruments and Opto-Electronics Engineering, Tianjin University, Tianjin 300072, China; yifanli@tju.edu.cn (Y.L.); yuyu1990@tju.edu.cn (Y.Y.); chenzl@tju.edu.cn (Z.C.); jlfking@tju.edu.cn (L.J.); mingxuancao@tju.edu.cn (M.C.); jqyao@tju.edu.cn (J.Y.); 2 Tianjin Key Laboratory of Low Dimensional Materials Physics and Preparing Technology, School of Science, Tianjin University, Tianjin 300072, China; htdai@tju.edu.cn

**Keywords:** three-dimensional graphene foams, broadband phototransistor, photoresponsivity, response time

## Abstract

Three-dimensional (3D) cross-linked polymer-like reduced graphene oxide foams (rGOFs) with a seamlessly continuous graphene network, exhibit high photoresponsive and conductivity and have received much attention regarding solar cells and supercapacitors. However, little attention has been paid to photodetection applications of 3D rGOFs. Here we report a novel broadband phototransistor based on metal-3D GFs-metal, which exhibits a high light absorption and a wide spectra response ranging at least from 400 to 1600 nm wavelength with a maximum photoresponsivity of 10 mA/W at 400 nm. In particular, stable and reproducible photocurrent cycles are achieved under different light blue light (405 nm), green light (532 nm), and NIR (808 nm) irradiations. Moreover, the device displays a typical transistor characteristic with a rapid response time of 18 ms at under 532 nm irradiation. The excellent performances indicate 3D rGOF as a promising candidate for future photodetection application.

## 1. Introduction

Graphene consisting of a sp^2^ hybridized C–C bonds in a honeycomb-like structure, exhibits unique optoelectronic characteristics, such as high carrier mobility, and superconductivity [1,2], which can potentially be applied in many applications, such as optical communication, biomedical imaging, night-vision and remote sensing [3,4,5,6,7,8]. Recently, single-, bi-, and tri-layer graphene phototransistors have been reported [9,10,11,12,13,14,15,16,17], which reveal a strong photoresponse within the metal and graphene channel. In addition, they also show a strong interband transition as compared to other materials [18]. However, difficulties in producing high-quality large-scale graphene, relatively low photoresponse current and weak absorption characteristics in most studies limit their application in the photodetection field [12,14,19,20]. For example, Fengnian Xia and his group have reported a maximum photoresponsivity of ~0.5 mA/W at a bias of 80 V using a 1.55 μm laser for (single and few-layers) graphene photodetectors [20]. In graphene-based photodetectors, the low photoresponsivity is mainly attributed to the low optical absorption in monolayer graphene and the short recombination lifetime (on the scale of a picosecond) of the photo-generated carriers [21].

Three-dimensional cross-linked polymer-like graphene foams (3D rGOFs), obtained from graphene oxide (GO), provide graphene materials with lower contact resistance, better conductivity, specific surface areas, stronger mechanical strengths and faster electron transport than single-, bi-, and tri-layer graphene, owing to the combination of the 3D porous structures and the excellent intrinsic properties of graphene [22,23,24]. Compared to the commercial photodetectors, which are fabricated with the gallium nitride (GaN), silicon (Si) and indium-gallium-arsenide (InGaAs) just applied to sensing ultraviolet (UV), visible and near-infrared (NIR) spectral regimes, respectively, the broadband, tunable optical absorption and high carrier mobility properties of 3D reduced graphene oxide foams make them promising materials for photodetectors. Several effective approaches have been developed to synthesize 3D graphene including: hydrothermal reduction method using graphene oxide (GO) solution, self-assembly in solution, chemical vapor deposition (CVD)-grown graphene foams, template-directed method [7,23,25,26]. In current developments, hydrothermal reduced graphene oxide (rGO) is widely used to synthesize 3D rGOFs because of the low cost and large-scale production [23,25,27]. Due to the unique properties and easy synthetic method, 3D rGOFs have attracted much attention for the study of solar cells, supercapacitors and electrodes, but rare attention has been paid to photodetector applications [25,28].

In this work, we demonstrate an Au/3D rGOF/Au structure phototransistor by using high-quality and large-scale 3D rGOFs. The device exhibits a high absorbance and high photoresponsivity in a broadband spectra, ranging at least from 400 to 1600 nm wavelength with a maximum photoresponsivity of 10 mA/W and a minimum of 5.6 mA/W, which is a 10-fold improvement compared to 0.5 mA/W of previous studies [12,20]. In particular, the device presents a high responsivity of 6.9 mA/W and a rapid response speed of 18 ms at 532 nm laser illumination. Moreover, the phototransistor displays stability and reproducibility of ON/OFF photoresponse cycles under different wavelength lasers, with blue light (405 nm), green light (532 nm), and NIR (808 nm) irradiations. Compared to commercial Si, InGaSn and ultrafast Ge photodetectors with the photoresponsivities of 0.52, 0.85, 0.85 A/W respectively and the response times of 5.9 µs, 160 ns, 100 ps, respectively, our devices need to be greatly improved. The results open up a new avenue for studying broadband and ultrafast detectors and reveal that 3D rGOF phototransistors have a great potential in numerous applications related to photodetection.

## 2. Materials and Methods

The 3D rGOFs supplied by Yongsheng Chen group Nankai University were synthesized by hydrothermal reduction method using graphene oxide (GO) solutions, following the optimized the synthetic method reported before [23,25]. The graphene oxide (GO) was prepared by using a modified Hummers method. After differential centrifugation, the GO solution (1 mg mL^−1^) was thermally treated in a custom Teflon-lined autoclave at 180 °C for 12 h. To obtain the 3D rGOF material, the three dimensionally cross-linked graphene needs to be dried in a vacuum oven at 100 °C for 2 h. After that, the obtained 3D rGOFs were prepared by a laser cutting machine (Guohong Machine tool, Botou, China). Then, the as-prepared 3D rGOF samples were carefully transferred on Si^n+^/SiO_2_ substrates which were treated in a UV ozone system (Mvcro, Beijing, China) for 15 min. The Si^n+^ was n doped silicon wafer. The electrodes were thermally evaporated through a shadow mask on the 3D rGOF with Cr/Au (10/200 nm) under the vacuum of 3.0 × 10^−4^ Pa.

The surface structure of 3D rGOFs was observed by a scanning electron microscope (SEM, Hitachi, Tokyo, Japan). The Raman spectroscopy was performed with a RENISHAW laser Raman spectrometer (Renishaw, London, UK). The broadband spectrum response and absorption spectrum of 3D rGOF were measured by a Zolix Omni-λ 3007 spectrophotometer (Zolix, Beijing, China) with Si and InGaAs photodetectors. For electric measurements, *I*–*V* characteristics and photoresponse current between the source (at ground) and drain electrodes of the 3D rGOF were tested by a Keithley 2400 with Labview software (Keithley, Beaverton, OR, USA) and the gate electrode was connected with a constant voltage source HP6030A. In this work, the light sources were 405, 532, and 808 nm semiconductor lasers.

## 3. Results and Discussion

### 3.1. Device Fabrication and Material Characterizations 

**Figure 1:**
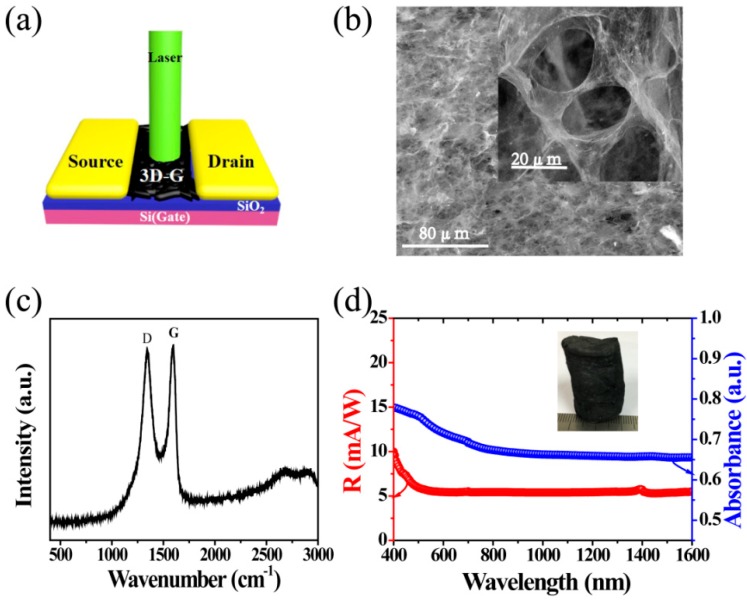
(**a**) Device schematic diagram; (**b**) SEM images of the 3D reduced graphene oxide foam (rGOF); (**c**) Raman spectra of the 3D rGOF; (**d**) The photoresponsivity (R) and optical absorption spectrum of the device as a function of wavelength ranging from 400 to 1600 nm. (Inset: picture of 3D rGOF columnar).

The architecture of the 3D rGOF phototransistor is fabricated with the configuration model shown in Figure 1a. The device includes a highly doped silicon wafer (Si^n+^) with a 300 nm thick gate layer, a SiO_2_ (capacitance C_ox_ of 11.5 nFcm^−2^) gate dielectric layer, a 3D rGOF active layer and source and drain electrodes. In particular, the 3D rGOF was synthesized by hydrothermal reduction method, and the source and drain (Au-Au) electrodes were thermally evaporated through a shadow mask, with channel length *L* = 3 mm and channel width *W* = 2 mm, respectively. Figure 1b exhibits the surface scanning electron microscopy (SEM) image of the 3D rGOF device with a scale of 80 μm and the insert shows the surface morphology with a scale of 20 μm. The 3D rGOF shows across-linked and sponge-like structure with a pore diameter about 20 μm [27,29,30,31,32]. The typical Raman spectrum of the 3D rGOF is shown in Figure 1c. Two prominent characteristic peaks at ~1341 and 1592 cm^−1^ correspond to the graphene band D and G [29,33], and the absence of 2D peak means no layer accumulate. Figure 1d shows the photoresponsivity and absorption spectrum of the device at wavelength ranging from 400 to 1600 nm. The broadband absorption spectrum of 3D rGOF express a 65%–80% absorbance (which is affected by material defects) at least from 400 to 1600 nm wavelength indicating that this device can be used for broadband spectra detection. The wide absorption spectra are due to the unique structure of the zero-band gap between the valence band and conduction band of 3D rGOFs [25,34]. Moreover, the device exhibits a broadband photoresponse (400−1600 nm) with a maximum R of 10 mA/W and a minimum of 5.6 mA/W under 0 V source-drain voltage and 0 V gate voltage. Compared to the (single and few-layer) graphene photodetector with a maximum photoresponsivity of 0.5 mA/W reported by Fengnian Xia’s group, the 3D rGOF phototransistor revels about an order of magnitude improvement of the photoresponsivity [20]. The high responsivity can be related to the higher electrical conductivity, faster spatial separation and transportation of photogenerated charge carriers and higher absorbance, as compared with single and few-layers graphene. The insert is a photo of the columnar 3D rGOF.

### 3.2. Electrical Characteristics

**Figure 2:**
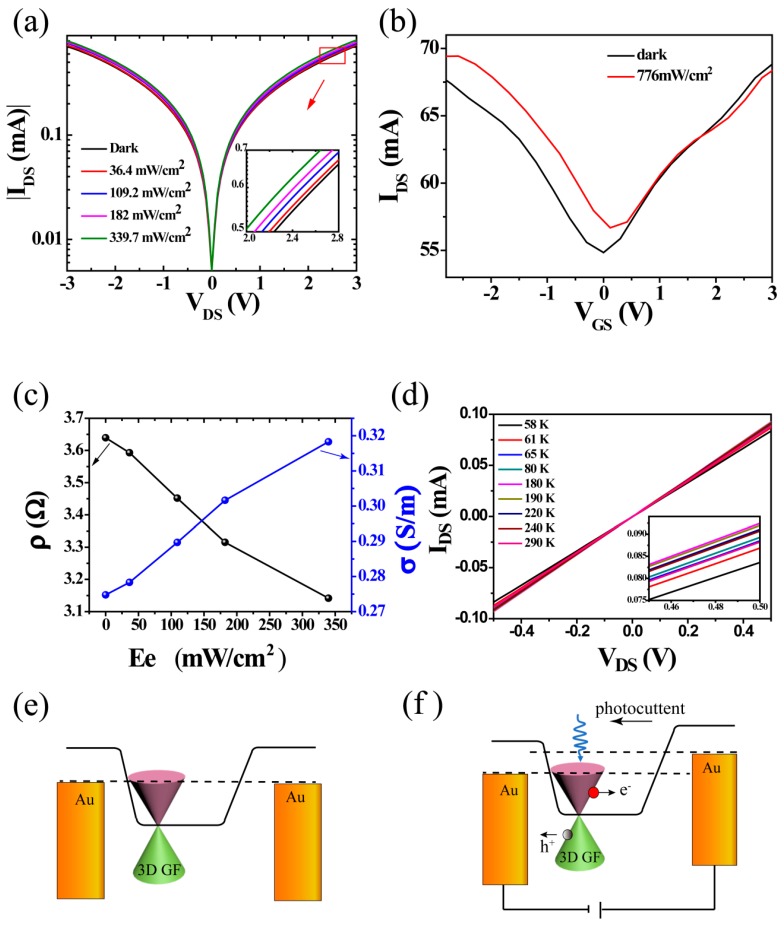
Electric properties of the 3D rGOF phototransistor. (**a**) Output characteristic (*I_DS_*–*V_DS_* at *V_GS_* = 0.3 V) under 532 nm laser; (**b**) Transfer characteristics (*I_DS_*–*V_GS_*) of the device under light condition or not at *V_DS_* = 0.1 V, respectively; (**c**) Resistivity (ρ) and conductivity (σ) of the device as a function of laser irradiance under 1.2 V bias voltage; (**d**) Temperature dependent *I*–*V* curves of the device between 58 and 290 K. Insert: temperature dependent *I*–*V* curves of the device between 58 and 290 K as a function of 0.45 to 0.5 V bias voltage; (**e**,**f**) Fermi energy and photocurrent generation mechanism model of the 3D rGOF phototransistor with and without light illumination and bias voltage.

For electric measurements, *I*–*V* characteristics and photoresponse current between the source (at ground) and drain electrodes of the 3D rGOF were tested. Figure 2a describes the *I_DS_*−*V_DS_* characteristics as a function of the source–drain bias under dark and with (36.4, 109.2, 182, 339.7 mW/cm^2^) 532 nm light illumination at a fixed gate bias. As shown in Figure 2a, *I_DS_* increases with the increase of *V_DS_*. Moreover, the device displays a sensitive photoresponse under weak light irradiation and an increased photoresponse with light illumination intensity raised from low to high. The insert shows how the photo current varies with *V_DS_* from 2.0 V to 2.8 V. It is clearly seen that the photoresponse current increases with the increasing illumination intensity and under the same light irradiation the current increases with the increasing source-drain voltage. It can be understood that the photo induced carriers are enhanced by increasing bias voltage, which is ascribed to the increase of the electron and hole concentrations. The photocurrent generation is based on the separation of electron–hole (e–h) pairs 3D rGOF induced in 3D rGOF by external electric fields. The 3D rGOF phototransistor reveals a high photocurrent of 0.1 mA under 2.8 V bias voltage [19,20]. Figure 2b shows transfer characteristics of the 3D rGOF phototransistor with a bias voltage *V_DS_* = 0.3 V. In the transfer curves, the device exhibits a “V” shaped transfer curve and typically ambipolar characteristics reveal either electrons or holes transport in the n-type or p-type transport regions of the device, respectively [9,35]. As shown in Figure 2b, the curve shifts toward positive *V_GS_* with light illumination indicating a typical p-type doping [36], which means that electrons move from 3D rGOF to Au electrode owing to the difference of electron potentials and the holes tend to remain in the 3D rGOF. Figure 2c shows the resistivity(ρ) and conductivity(σ) of the device as a function of laser irradiance under 1.2 V bias voltage. It shows that with the laser irradiance increasing the resistivity decreases but the conductivity increases, which can be explained by the photoconductivity mechanism. With the increasing of the laser intensity, the free carriers increase leading to a decreased resistance to charge migration, which results in the decreased resistivity and increased conductivity. The potential variation and Fermi level mechanism for the photocurrent generation are shown schematically in Figure 2d. When light illuminates the 3D rGOF, electron–hole (e–h) pairs generate at the 3D rGOF interface between the electrodes and 3D rGOF. To exclude potential contributions from thermal effects, we measured the *I_SD_*–*V_SD_* at temperatures from 58 to 290 K. Figure 2d shows the temperature dependent *I*–*V* curves of the device between 58 and 290 K. As illustrated in the figure, the current increases with an increasing temperature from 58 to 180 K, and a slight decrease in the range 180–290 K. The temperature dependent *I*–*V* curves of our device are consistent with the report results [37]. The small temperature dependence of *I_SD_*–*V_SD_* allows us to exclude the temperature effect caused by laser illumination. Therefore, the photoresponse of our device is ascribed to a photoconduction mechanism. In Figure 2e,f, the solid line presents the Fermi energy of the two Au electrodes under light illumination. The e–h pairs become separate and a photocurrent is generated under the application of an external field. Usually, the recombination time of the e–h pairs is tens of picosecond, which is due to the quality and carrier concentration of the 3D rGOF [11,19,20]. Therefore, photocurrent generation is based on the separation of the e–h pairs under an external field. Moreover, in the 3D rGOF photodetectors, the honeycomb network in 3D rGOF allows for the almost unimpeded transmission of carriers through the potential barriers, leading to high-bandwidth photodetection.

### 3.3. Photoresponse Characteristics

**Figure 3:**
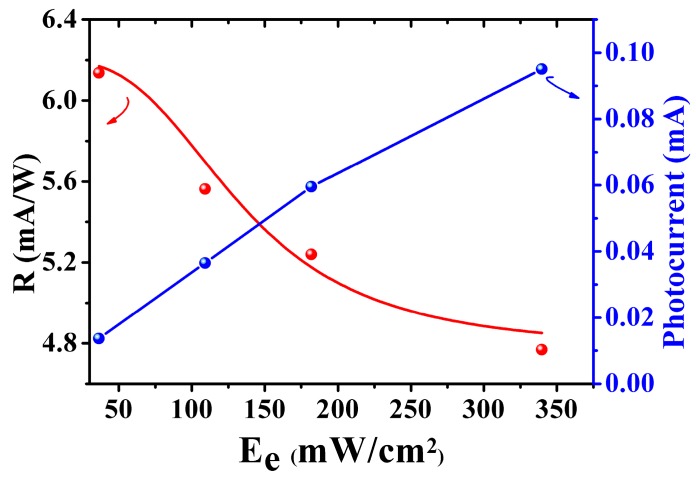
The photoresponsivity (*R*) and photocurrent of the device at 532 nm as a function of *Ee*.

For the 3D rGOF photodetectors, photoresponsivity (*R*) is a key parameter [38,39,40]. Figure 3 displays the photoresponsivity (*R*) and photocurrent of the device at 532 nm as a function of laser irradiance *Ee*. It shows that the photocurrent increases with the increasing *Ee* and the maximum photoresponsivity (*R*) is 6.9 mA/W under 2.4 V bias voltage calculated by Equation (1).

(1) $$R=\frac{\Delta I_{DS}}{P}=\frac{I_{illu}-I_{dark}}{E_{e}\times S}$$

*I_illu_* and *I_dark_* represent the drain current under illumination and in the darkness, respectively. *P*, *Ee*, and *S* are power density, irradiance which means the radiation energy per unit area, and the effective channel area, respectively. According to the previous studies [38,39], increasing *Ee*, *R* decreases as a reciprocal function of this type $R=a/\left[1+{\left(E_{e}/b\right)}^{n}\right]$: where *a* and *b* are constants, and *n* is a fitting parameter. Therefore, we introduce Equation (2) in this work.

(2) R=(ehνT0Tr)11+(PP0)n

where *e* is the charge of a single electron, *hυ* is the single photon energy, *T_0_* is the carrier lifetime, *T_r_* is the carrier transit time, *P_0_* is the excitation intensity for which the surface states are fully filled, and *n* is the fitting parameter. With a proper choice of the parameters, the red solid line (fitting line) fits well the experimental data.

**Figure 4:**
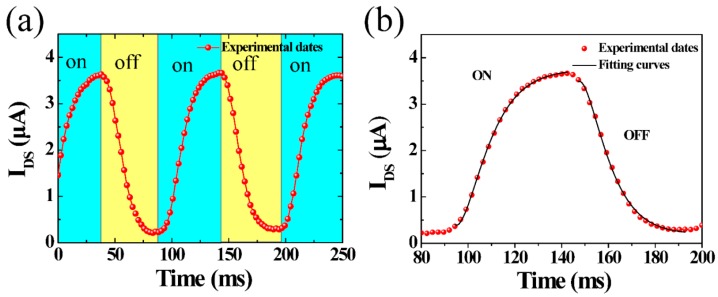
Photoresponse characteristics of 3D rGOF phototransistor with 532 nm laser under 50 mW/cm^2^ irradiation with *V_DS_* = 0.01 V, *V_GS_* = 0 V; (**a**) Current response cycles of the device (**b**) Time-dependent photocurrent responses during the rise and fall phases.

Photoresponse speed is another key characteristic for a photodetector. In this work, a chopper was used to switch the light ON/OFF and a oscilloscope was used to acquire the photoresponse currents. Figure 4a shows that the device exhibits stable and reproducible ON/OFF switch photocurrent responses with light irradiance of 50 mW/cm^2^. The photoresponse current increases once the light turns on and then decreases as the light turns off within 20 ms, respectively. This indicates that the photogenerated carriers increase with the increasing light illumination, which allows easier charge tunneling and transportation than in darkness. Figure 4b displays the time response of photocurrent rise and fall for the3D rGOF phototransistor in response to 532 nm illumination at irradiance of 50 mW/cm^2^ under 0.01 V bias voltage and 0 V gate voltage. As soon as a hole reaches the drain, another hole replenishes the supply from the source. According to the photoconductive relaxation theory [18,40], the photoresponse current time of the 3D rGOF phototransistor can be well described by the Equation (3) and (4) for growth and decay, respectively.

(3) $$I\left(t\right)=I_{dark}+A\left[\exp(t/\tau_{1})\right]$$

(4) $$I\left(t\right)=I_{dark}+A\left[\exp(t/\tau_{2})\right]$$

where *I_dark_* is the dark current, τ is the time constant, t is the response time when 532 nm laser is switched on and off, *A* is a scaling constants. Figure 4b shows that the fitting curves (solid line) match the experimental data (red dashed lines) well. The photocurrent increases in about 18 ms at a 1/2 time value before saturating. The photocurrent drop trend starts with a fast decay in the first 5 ms at a 1/2 time value after the light turned off and a slow photocurrent decay process lasts about 17 ms at a 1/2 time value before the current decreasing, reaches the dark current value. The fast photoresponse speed displays a huge improvement compared to the photoresponse speed (26 s) reported by N. N. Rao’s group [18].

### 3.4. MultibandPotoresponse

**Figure 5:**
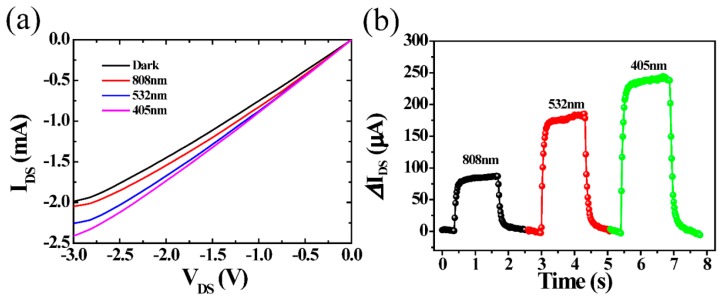
(**a**) *I_DS_*−*V_DS_* output characteristics at the incident power of 26 mW under various illuminations of 405, 532, and 808 nm, with *V_GS_* = 3 V; (**b**) Photoresponse current at the incident power of 26 mW with various illuminations of 405, 532, and 808 nm under *V_DS_* = −1.5 V and *V_GS_* = 3 V.

According to Figure 1d, 3D rGOF exhibits broad absorption and photoresponse ranging from 400 nm to 1600 nm. In order to investigate wide spectral response, the dark currents and photocurrents are measured by varying the range of laser wavelengths under the same irradiation. *I_DS_*−*V_DS_* output characteristics with different wavelength (405, 532, 808 nm) at the incident power of 26 mW under *V_GS_* = 3 V are shown in Figure 5a. It shows that photocurrents increase with the increasing bias voltage under the wavelength of 405, 532, 808 nm, respectively. Figure 5b shows that the phototransistor exhibits stability and reproducibility in the progress of ON/OFF cycles under different wavelength illumination at −1.5 V bias voltage. The photocurrents excited by 405, 532, and 808 nm light are ~240, ~180, ~80 μA, respectively. Due to the different photoelectric conversion efficiency and the absorbance of different wavelengths, the photocurrent value is higher for shorter wavelengths than for longer ones, consistent with the absorption spectrum of the device, which is the usual case for phototransistors [37,38,39,40,41].

## 4. Conclusions

In summary, we have fabricated a broadband and fast phototransistor based on 3D rGOFs. The interaction of photon and 3D rGOF and the generation of photo-generated carriers have been demonstrated. The device exhibits a high absorbance and broadband spectral response in the wavelength range at least from 400 to 1600 nm with a maximum photoresponsivity of 10 mA/W. The performances of phototransistor are measured under 532 nm and display a maximum photoresponsivity of 5.9 mA/Wand a rapid response of ∼18 ms. Moreover, the device presents a stable and reproducible behavior in the progress of ON/OFF photoresponse cycles under the illumination of laser with different wavelength UV (405 nm), visible (532 nm), and NIR (808 nm). The results prove the potential of 3D rGOFs as a promising material for efficient optoelectronic devices.
